# Editorial: New perspectives in glaucoma pathophysiology, diagnosis, and treatment

**DOI:** 10.3389/fmed.2023.1200427

**Published:** 2023-05-03

**Authors:** Alessio Martucci, Carlo Nucci, Maria Dolores Pinazo-Duran

**Affiliations:** ^1^Ophthalmology Unit, Department of Experimental Medicine, University of Rome “Tor Vergata”, Rome, Italy; ^2^Ophthalmic Research Unit “Santiago Grisolia”, Foundation for the Promotion of Health and Biomedical Research of the Valencian Community, Valencia, Spain

**Keywords:** glaucoma, retinal ganglion cells (RGCS), visual field, quality of life, glaucoma diagnosis, glaucoma pathophysiology, glaucoma treatment

Glaucoma is the most common cause of legal blindness worldwide. This progressive optic neuropathy is characterized by the degeneration of retinal ganglion cells and consequent changes in the optic nerve head anatomy.

The prevalence of the different forms of glaucoma varies on basis of age, gender, ethnicity, and economical levels.

People with glaucoma, initially experience peripheral vision loss, and if not treated promptly, vision loss may get worse, possibly leading to blindness over time. The disease is extremely complex, and its pathogenesis is still not fully understood. Although intraocular pressure is considered the major cause of retinal ganglion cell death, glaucoma can even occur if intraocular pressure is within the normal range. In these cases, other risk factors such as abnormally low cerebrospinal fluid pressure, impaired microcirculation, mitochondrial deficits, altered immunity, excitotoxicity, and oxidative stress or coexisting ocular or systemic diseases may also concur to the development and the progression of the disease (1).

Genetics may also play a role in the disease. An increasing body of evidence suggests the presence of susceptibility loci in the DNA. However, the mechanism by which these genes might contribute is not clear.

Due to the disease's complexity, early glaucoma diagnosis can be challenging for clinicians. Visual field examination provides late information as damage is expected when at least 30% of retinal ganglion cells have been damaged. Hence the need for new diagnostic tools that allow an easier and earlier diagnosis of the disease (2).

Once diagnosed, glaucoma treatment may be challenging for clinicians. By now, intraocular pressure reduction is the primary strategy to treat glaucoma. However, some patients may not respond to the treatment hence the need for new intraocular pressure lowering molecules or different therapeutic approaches that may comprehend neuroprotection, systemic intervention, para surgical or surgical intervention (3).

In this Research Topic, 14 original research articles examined several topics including the prevalence of glaucoma, new pathophysiological insight, including psychometric aspects and impact on quality of life, as well as new options for diagnosis, treatment, and overall management of this complex disease.

One paper described the characteristics, epidemiology, management, and outcomes of glaucoma in pediatric patients in central China. Liu Q. et al. reported that primary congenital glaucoma, juvenile open-angle glaucoma, and traumatic glaucoma turned out the most prevalent subtypes of pediatric glaucoma in central China.

Six papers evaluated novel biomarkers or diagnostics tools useful for the early diagnosis of glaucoma. Lillo et al. showed that glaucoma, even when the ocular pressure is pharmacologically kept under control, causes significant variation in the level of the compounds of the aqueous humor which are also essential for mitochondrial function. In this regard, Pinazo-Duran et al. reported that the development of composite biomarkers from tears, aqueous humor and blood, seems to be an appropriate solution in ophthalmological practice for early diagnosis and to predict therapeutic response in glaucoma patients.

Yuan et al. analyzed the role of anthropometric aspects in glaucoma. Herein, the Mendelian randomization method revealed that increased body mass index and waist circumference are potential risk factors for glaucoma.

Yang et al. evaluated the usefulness of optic disc photographs for morphologic features and glaucoma likelihood, showing that the stereoscopic method was superior compared to the monoscopic method for general ophthalmologists.

In this context, Seo et al. highlighted that deep learning models may be the future of diagnostic tools. Convolutional neural network models have been shown to be able to discriminate early normal-tension glaucoma from glaucoma suspect eyes using Bruch's membrane opening-based optic disc photography.

This new disc photography of Bruch's membrane opening overview can aid in the diagnosis of early glaucoma.

Besides, increasing evidence suggests that glaucoma is a neurodegenerative disease that originates in the brain but manifests as an eye disease (4–7). Liu K. et al. showed the usefulness of the Mendelian randomization tool to understand the causal effect of brain alterations on glaucoma. Thus, supporting the rising evidence of the existence of the brain-eye axis.

Three articles dealt with the therapeutic aspect of glaucoma. Martucci et al. proposed the combined use of neuroprotectors, such as citicoline and Coenzyme Q10, due to their putative synergistic effect and combined action on the different pathogenetic targets causing the onset and progression of glaucoma. Using combined treatment may downregulate more pro-apoptotic pathways and boost the effect on one or more pathways on which the different molecules act.

Besides, some patients need special care. In the case of moderate glaucoma, Wang et al. proposed ultrasound cyclo-plasty as a safe and effective method for reducing IOP. While, according to Li et al., patients who have undergone keratoplasty, are more likely to develop glaucoma before surgery. In these cases, surgical treatment should be selected according to the ocular surface condition to reduce the occurrence of complications.

Psychological factors may also interfere with the treatment. Two papers on this topic focused on this aspect. A case-control study by Chu et al. documented that IOP was significantly reduced in patients with open-angle glaucoma after “planning” selective laser trabeculoplasty treatment, even without actually performing it.

Peng et al. underlined the importance of resilience as a positive factor, which could improve patients' quality of life with glaucoma. However, other factors such as sleep disturbance may reduce this positive impact. Thus, supporting the importance of interventions that enhance the levels of resilience and promote healthy sleep in glaucoma patients.

As seen, studies on primary open-angle glaucoma extend well beyond ophthalmology to biochemistry molecular biology, general internal medicine, pharmacology, pharmacy, science technology, and other areas. Two papers focused on the importance of the literature analysis also using the emerging techniques of artificial intelligence. Zhao et al. conducted a bibliometric analysis of publications on glaucoma from 2000 to 2021 in the Web of Science database. Thus, suggesting that the analysis of co-occurrence networks provides researchers with information about potential collaboration opportunities with other institutions and researchers. Bibliometric analyses provide valuable guidance for researchers in the selection of Research Topics. In this contest, AlRyalat et al. proposed that with the emergence of artificial intelligence and machine learning techniques that seek to decipher new learnings from these large datasets, a deep and nuanced understanding of the designs and methodologies used may be key to unlocking even more data to enhance patient care.

In summary, this Research Topic focuses on new insights into the disease prevalence and pathophysiology, on the development of a new generation of diagnostic tools and biomarkers, and on new treatments scheme that may assist ophthalmologists in the early care of their patients, preserving their quality of life.

## Author contributions

All authors listed have made a substantial, direct, and intellectual contribution to the work and approved it for publication.
